# Factors Influencing Household Uptake of Improved Solid Fuel Stoves in Low- and Middle-Income Countries: A Qualitative Systematic Review

**DOI:** 10.3390/ijerph110808228

**Published:** 2014-08-13

**Authors:** Stanistreet Debbi, Puzzolo Elisa, Bruce Nigel, Pope Dan, Rehfuess Eva

**Affiliations:** 1 Department of Public Health and Policy, Institute of Psychology, Health and Society, Whelan Building, University of Liverpool, Liverpool L69 3GB, UK; E-Mails: puzzoloe@liv.ac.uk (P.E.); ngb@liv.ac.uk (B.N.); danpope@liv.ac.uk (P.D.); 2 Institute for Medical Informatics, Biometry and Epidemiology, University of Munich, Marchioninistr. 15, Munich 81377, Germany; E-Mail: rehfuess@ibe.med.uni-muenchen.de

**Keywords:** household air pollution, solid fuel use, improved stoves, scale up, adoption, developing countries

## Abstract

Household burning of solid fuels in traditional stoves is detrimental to health, the environment and development. A range of improved solid fuel stoves (IS) are available but little is known about successful approaches to dissemination. This qualitative systematic review aimed to identify factors that influence household uptake of IS in low- and middle-income countries. Extensive searches were carried out and studies were screened and extracted using established systematic review methods. Fourteen qualitative studies from Asia, Africa and Latin-America met the inclusion criteria. Thematic synthesis was used to synthesise data and findings are presented under seven framework domains. Findings relate to user and stakeholder perceptions and highlight the importance of cost, good stove design, fuel and time savings, health benefits, being able to cook traditional dishes and cleanliness in relation to uptake. Creating demand, appropriate approaches to business, and community involvement, are also discussed. Achieving and sustaining uptake is complex and requires consideration of a broad range of factors, which operate at household, community, regional and national levels. Initiatives aimed at IS scale up should include quantitative evaluations of effectiveness, supplemented with qualitative studies to assess factors affecting uptake, with an equity focus.

## 1. Introduction

### 1.1. Traditional Household Energy Practices as a Major Health and Development Problem

Ensuring access to clean and efficient household energy is one of the major challenges faced by developing and middle-income countries today. Approximately 2.8 billion people rely on solid fuels, including biomass (e.g., wood, dung, crop residues, charcoal) and coal, to meet their cooking and heating needs [1]. These solid fuels are typically burnt in traditional, inefficient stoves causing high levels of household air pollution (HAP), considerably higher than WHO recommended levels for particulate matter [2], and frequently breaching guidelines for carbon monoxide and other pollutants [3].

Traditional household energy practices have dramatic consequences for health, the environment and socio-economic development. HAP from burning solid fuels is an important risk factor for child pneumonia [4], chronic respiratory diseases [5] lung cancer in adults [6], adverse pregnancy outcomes [7] and several other health outcomes including cataracts [8]. HAP is ranked highly in the Global Burden of Disease and was associated with 3.5 million annual deaths, plus a further 0.5 million due to ambient HAP, and 4.3% of disability-adjusted life years in the year 2010 [9]. The most vulnerable group for HAP are women and children [10]. Time spent collecting biomass fuel and cooking can impact negatively on education and development [11]). Where fuel cannot be collected, a disproportionate amount of household income is spent on purchasing it. Lack of access to modern energy therefore contributes to trapping poor households in a cycle of ill-health and poverty [10]. The inefficient burning of solid fuels also represents an unsustainable use of natural resources, contributing significantly to the release of climate pollutants [12].

### 1.2. Global Commitment to Promoting Access to Cleaner and More Efficient Household Energy

Several global [13,14,15] and regional initiatives [16,17] have emphasised the need to address this crisis. In view of growing action and substantial untapped financial resources in development aid, private sector investment and official/voluntary carbon offset schemes, the large-scale promotion of modern household energy seems more realistic today than ever before.

A number of cleaner alternatives to traditional cooking practices are available including a range of improved solid fuel stoves (IS) and cleaner fuels. Among middle-income households in developing countries and middle-income countries, gas, and in particular liquefied petroleum gas (LPG), has already replaced solid fuel for all or selected cooking tasks. However, it is relatively expensive compared to solid fuel and lack of supply to many rural areas is a limiting factor. In selected settings, biogas, alcohol stoves and solar cookers can also provide an efficient and clean source of household energy, but they tend to be “niche solutions”, and are less likely to be scaled up globally. Thus, solid fuels are likely to continue to be utilised among the poorest households in developing countries, and therefore have an important role to play in global access to cleaner household energy, at least in the short to medium term.

There has been considerable research into the effectiveness of IS in reducing HAP, [18,19,20,21,22] but little focus on successful approaches to disseminating these interventions at scale [23]. Such evidence is of particular importance to achieving the “quantum leap” [10] required for sustained adoption at scale and meet targets set by the Global Alliance for Clean Cookstoves to foster the adoption of clean stoves and fuels in 100 million households by 2020 [13]. Lewis and Pattanayak [23] carried out a systematic review of factors affecting adoption of IS and clean fuels which identified a number of factors relevant to adoption, but the review included only quantitative studies and does not offer any explanation of the likely mechanisms that underlie these associations, so it is difficult to draw conclusions with respect to the development of programmes and policies. In addition, an understanding of household views on these stoves is vital if “scale up” is to be effective in the communities targeted. However, to date, there has been no attempt to collate such insights using systematic review methodology.

### 1.3. Objective of This Review

This qualitative systematic review aimed to provide an in- depth analysis of the views held by stove users and other stakeholders on different factors that influence the large-scale uptake by households of IS in low- and middle-income countries. The review includes analysis of all factors that are likely to impact on household decisions to adopt IS, whether these operate at a household, community, regional or national level.

## 2. Materials and Methods

The qualitative systematic review was embedded in a wider review, which included IS as well as LPG, biogas, alcohol fuels, and solar cookers and incorporated qualitative, quantitative and case studies [24]. This paper reports the findings of the qualitative review, which is based on only qualitative studies and pursued a qualitative methodology, providing in-depth insights of the perceptions of actual and potential users of improved stoves and other stakeholders involved in building, marketing or selling improved stoves. It also reports the detailed findings of the review in relation to equity. The review was registered with the Evidence for Policy and Practice Information and Co-ordinating Centre (EPPI-Centre) at the University of London and a detailed, peer-reviewed protocol is available [24].

### 2.1. Inclusion and Exclusion Criteria

Solid fuels are used for cooking, heating, lighting, boiling water and home-based income-generation. Since heating is highly climate- and season-specific, this review focused on cooking as the most important use of solid fuels worldwide but also considered the importance of IS in meeting other household energy needs. It included projects or programmes promoting IS among households, but excluded those directed at public or commercial settings and refugee camps. The review was concerned with IS initiatives undertaken at any scale, attempting to learn about adoption (acquisition and initial use of less than one year) and sustained use (more than one year after acquisition).

Studies relating to a direct experience with IS, providing empirical information on the experience of either household users or those producing and disseminating IS, conducted in urban and rural settings of low- and middle-income countries [25] were included. Studies were eligible if they collected and analysed data using qualitative methods, either as a stand-alone study or as part of a mixed-methods approach.

### 2.2. Search Strategy

A systematic search of published and unpublished sources of evidence was undertaken utilising over twenty peer-reviewed databases across multiple disciplines, and grey literature searches, as well as hand searches of references of included studies [24]. We also included unpublished literature provided by key experts in the field and relevant results identified through Google and Google Scholar. Various *intervention* search terms were combined with *uptake* search terms using the Boolean operator “AND”, making use of pluralisation and wild cards (see Table 1) and adapted to the needs of specific databases. The search strategy aimed to identify all types of study designs for the purposes of the full review, and studies were subsequently allocated to either the qualitative, quantitative or case study categories as appropriate [24].

**Table 1:** Search terms used for the main search strategy on IS.

Intervention AND	Uptake
* stove/* stoves cook* AND technol* cook* AND fuel* chulha/chulhas chulla/chullas chullah/chullahs chulas	Adopt* accept* deliver* dissemin* implement* scale “scal* up” “roll* out” “tak* up” uptake

Studies published between 1980 (when programmes to promote fuel efficiency and protect biomass resources were becoming common) and June 2012 were included. In addition to English language papers, foreign language papers published in Spanish, Portuguese, French, German and Italian were also considered.

### 2.3. Screening Studies

Initial selection of studies, based on titles and abstracts, was conducted by one author, with independent verification of a random 20% of studies. Full papers were independently screened for relevance by two or more investigators, with all decisions for inclusion/exclusion being documented using the EPPI Reviewer 4 Software (http://eppi.ioe.ac.uk/cms/Default.aspx?alias=eppi.ioe.ac.uk/cms/er4). Disagreements were reconciled through discussion within the research team.

### 2.4. Data Extraction and Quality Appraisal

Data were independently extracted by two researchers onto data extraction forms including information on study setting, intervention, methodology, barriers and enablers. All papers were assessed for methodological quality using 11 established criteria adapted from Harden *et al.* [26] reflecting: (a) quality of reporting (*i.e*., study objectives, rationale, context, methods of data collection, data analysis and interpretation); (b) use of strategies to establish reliable data collection and analysis and to ensure validity of findings; and (c) the extent to which findings reflect participant perspectives and experiences. Studies were then classified as strong (9–11 criteria), moderate (5–8 criteria) or weak (1–4 criteria). Any discrepancies were resolved through discussion. Following the approach taken by Jensen and Allen [27] that suggests broad inclusion criteria afford the greatest understanding of the phenomenon and that studies should not be excluded on the basis of quality, this classification was not used as a criterion for post-hoc exclusion but as a basis for excluding weaker studies in a sensitivity analysis.

### 2.5. Evidence Synthesis

Initially we sought to identify factors influencing adoption (initial uptake) and sustained use (use over 12 months or longer) separately, but the majority of studies did not discriminate between the two, due to their short-term nature. Thematic synthesis was adopted to analyse the data [28]. Data reflecting the views of the participants and the interpretation of the study authors were included in the analysis. Data synthesis was carried out in three stages: (i) data were initially coded line by line by two investigators; (ii) codes were then combined to generate a set of descriptive themes for each study through discussion between two investigators; and (iii) analytical themes across studies were developed. Recording of the process of development of themes was explicit to ensure the methodology was transparent and rigorous.

To provide a structure for compiling the findings of the wider review, we identified and adapted domain areas highlighted in previous literature as being necessary for successful scale up [12,29]. This framework was then tested against the themes and factors identified in the literature, and was found to be a useful tool for the purpose, with no substantial revision being required. The framework includes seven domains: (1) Fuel and technology characteristics; (2) Household and setting characteristics; (3) Knowledge and perceptions; (4) Financial, tax and subsidy aspects; (5) Regulation, legislation and standards; (6) Market development and (7) Programmatic and policy mechanisms. Themes identified from the qualitative analysis were assigned to the appropriate domains and summarised in tabular and narrative form. All themes identified, mapped onto one of the seven domains, providing support for the comprehensiveness of the framework.

## 3. Results

### 3.1. Characteristics of Included Studies

**Figure 1:**
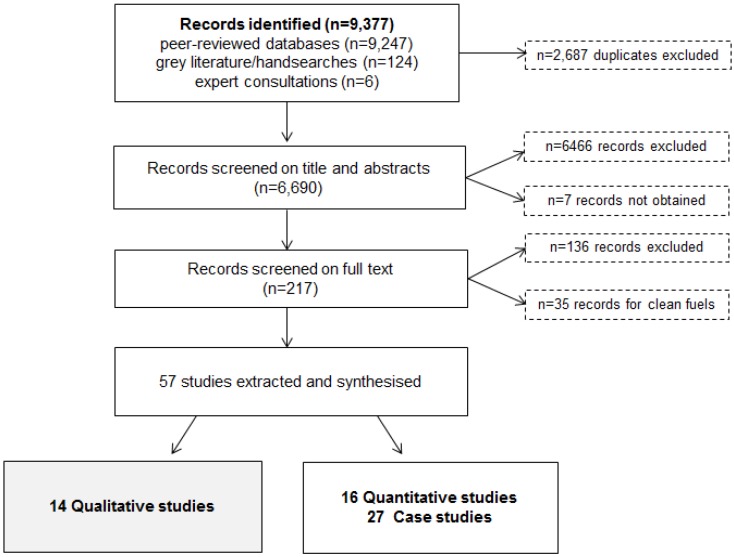
Flowchart of search results.

Figure 1 outlines the outcome of the search and screening process for the wider review. The overall search identified 9377 records; of these, fourteen were qualitative studies concerned with uptake of IS and therefore met the inclusion criteria for this review.

These fourteen studies were conducted in India (*n* = 4), Kenya (*n* = 2) Mexico (*n* = 4), Mongolia (*n* = 1), Nepal (*n* = 1) and Bangladesh (*n* = 2) (Table 2). Only one study was conducted in an urban area [30] and concerned use of coal, the rest were based in rural areas using biomass as the principal fuel. Only one study was conducted at national level [31] rather than community or regional level. IS types varied from simple self-constructed biomass stoves (e.g., clay and mud stoves) to more sophisticated stove designs (e.g., Patsari stoves) and improved coal stoves suitable for meeting cooking as well as heating needs. Study designs included semi-structured interviews (SSIs) (6 studies), key-informants interviews with stakeholders (KIIs) (3 studies), focus group discussions (FGDs) with stove users/non-users (6 studies), and participant observation (6 studies).

The majority of studies explored the perceptions of users and non-users (*n* = 13), with some studies also reporting the views of stove builders, [32,33], stove promoters [34,35] and project staff or other stakeholders [31,32,33,35]. The quality of the studies varied, with five studies assessed as strong, eight as moderate and one as weak (Table 2).

In the moderate and strong studies, context was explained clearly and the description of sampling and methods used to collect the data were clear. However, there was not always enough information given about data analysis or methods employed to increase validity and reliability. While there were some limitations in the amount of quotes presented, in most studies, these were sufficient to allow the reader to verify findings. Also, all moderate and strong studies incorporated methods for ensuring data were grounded in the views of the participants, and provided enough information to conclude that the findings reflected the views of stove users and other stakeholders.

The study identified as weak [18], did not present any of the qualitative data on which the findings were based making it more difficult to assess validity. It was also the only study to report the failure of a project which led to discontinued use of IS by the women. The authors concluded that this failure was primarily due to poor design and quality of the IS, and the Forest department failing to convince the community of its benefits. Despite these methodological limitations, there was no evidence to suggest that the findings should be considered untrustworthy and a sensitivity analysis showed that the majority of findings were supported by other studies in the review.

Overall, thematic synthesis led to the identification of 18 factors related to adoption and sustained use of IS and these are presented below under the seven framework domains.

### 3.2. Domain 1: Fuel and Technology Characteristics

#### 3.2.1. Fuel Savings

Significant fuel savings were considered a motivation for adoption of IS [18,30,32,34,36].

“*Our mud stove requires too much fuelwood, but this (improved) stove requires less wood, that is why we like this stove.*” [36].

The value attached to fuel savings varied greatly and in relation to proximity and availability of fuel and the risk of fuel collection. In areas where firewood was purchased, cost savings were a major benefit [30,34]. However, where no value was attached to the time taken to collect fuel, fuel savings were not a motivating factor [37].

“*I don’t care if I use too much and run out of fuel*!” [37].

One negative aspect associated with some IS was the inability to burn leaves or agricultural residues [18,38].

#### 3.2.2. Time Savings

Perceived changes in workload associated with IS varied, with some women reporting less work for the upkeep [30] and others reporting considerably more, including wood having to be dried, chopped and stored [18,30,31,33,37,39]. Time savings associated with cooking on multiple pot-holes, were considered an important advantage [18,31,32,34,36,40].

“*There are two mouths, so cooking is done quickly. [...] We can cook on one mouth and we can heat the water on the other mouth simultaneously*” [36].

In contrast, women stopped using the stove if cooking time was prolonged [38] or if the stove was considered unsafe and needed continual supervision to control fire risk [18].

Frequent maintenance and cleaning of the IS were considered problematic by some users and a reason for discontinuing use [30,37,38,39].

#### 3.2.3. Stove Design and Durability

Culturally appropriate stove design is important. Stove modifications by users resulting from unsuitable design features included enlargement of the entrance [37,39] and grate removal [36,40] compromising the efficiency of the stove.

“*There was a grate in the stove, but we removed it. Now the flames go to the pot directly*” [40].

Some households believed chimney stoves to be incompatible with thatched roofing increasing fire risk, and proving complex to set up [18].

Durability is also important. Frequent stove cracking or parts breaking [36,39,40] had an impact on satisfaction and sustained use. Also, proper installation and regular cleaning were seen as critical for appropriate functioning [37,39].

Women often reported using multiple cooking strategies to utilise the benefits of different stoves, for example, combining IS with open fires [32,41] or with LPG [37]. Women who used different technologies were more likely to try an additional method [37]. Reasons for multiple cooking strategies included not being able to cook traditional food on the IS [38], requirements for smaller pieces of wood, doubts about the ability of a smaller stove to cook efficiently [31], and IS being considered unsuitable for larger gatherings, since it can be difficult to cook food in larger quantities on the IS [37,38].

### 3.3. Domain 2: Household and Setting Characteristics

#### 3.3.1. Socio-Economic Status

Where communities were required to pay the full cost for the IS, price was an important barrier among poorer households [35,36] especially households not engaging in the labour market [34].

“*Women felt that people who engaged in paid labor were more likely to be able to purchase a cookstove, but that very few people were paid for work”* [34].

In poorer households, the benefits of fuel saving were reported as an important selling point.

“*High levels of poverty in the community mean that households have different priorities competing for scarce resources, and fuel saving is valued principally because it translates into cost savings*.” [35].

However, uptake was reported to be more successful among better-off communities [35,37]. Many programmes focus on reaching as many households as possible rather than on equitable distribution. Indeed, Troncoso *et al.* [37] suggest that according to Roger’s diffusion of innovation theory, it may be best to target early adopters with late adopters following suit when a critical mass of users has been reached [42].

#### 3.3.2. Gender Roles

Clearly defined gender roles within the household emerged, with women taking primary responsibility for cooking, cleaning and childcare [34,36,37], and men making decisions about changes to kitchen construction and IS purchase [34,35,36,37]. Some women were, however, able to pay for the IS using their savings, set aside for purchasing clothes or additional food [34].

“*[Women] can save a little from the food money each time and find themselves having a lot more than their husbands expect. […] I had saved up to buy shoes and decided to buy the cookstove instead*.” [34].

Also, negotiations supported by co-wives or mothers-in-law [34,37] could influence husbands’ decisions in favour of IS purchase, although opposition by the mother-in-law has also been reported as a barrier to purchasing a stove.

“*My mother-in-law was the one who negotiated it for me. She told my husband that the cookstove was really good and she would like us to install one in her kitchen and mine so that we would have an easy time for cooking*” [34].

**Table 2:** Characteristics of included studies.

Author/Year &Reference	Country, Area	Setting	Improved Solid Fuel Stove Type	Intervention Programme	Study Design *	Sampling and Participants Characteristics	Data Collection Period and Language Used	Data Analysis	Quality Appraisal ^#^
Anderson 2007 [36]	India, Western Maharashtra	Rural	Bhagyalaxmi stoves	ARTI’s program, with support from Shell Foundation (since 2003)	Ethnographic case study making use of FGDs, SSIs, KIIs and PO	3 FGDs (7–8 women each; users/non-users), 3 SSIs (user/non-users), KIIs (project coordinator and translator)	Two years after stove installation; interviews in Marathi	Editing analysis (*i.e*., hermeneutic approach)	Strong
Chowdhury *et al*., 2011 [18]	Bangladesh, Habigony region	Rural	Mud stoves	Nishorgo Support project (2009)	Face-to-face survey and one FGD	No. = 70 (users/non-users women) and 1 FGD with community members	January–February 2009; interviews in Bangla	Not stated; descriptive narrative	Weak
Christoff 2010 [39]	Mexico, State of Mexico	Rural	Patsari and Onil stoves	SEDESOL National program	FGDs	4 FGDs (9–14 each; women users only)	July–November 2009; Interviews in Spanish	Thematic analysis	Strong
Gordon *et al*., 2007 [30]	Mongolia, State capital	Urban	Coal stoves	World Bank program	FGDs	3 FGDs (8 mixed-gender each, users/non-users); 6 SSIs interviews with users/non-users	Not specified (1375 stoves given at the time of the study); interviews in Mongolian	Editing analysis (*i.e*., hermeneutic approach)	Strong
Jagoe *et al*., 2006 [43]	India, Bundelkhand region	Rural	Anandi and Sukhad stoves	ARTI’s program, with support from Shell Foundation (since 2003)	FGDs at baseline and follow-up	Separate FGDs with men and women, 11 FGDs at baseline and 8 FGDs at 12 moths follow up. 3 KIIs at baseline, 3 and 12 months	October 2004– September 2005; interviews in Hindi or local dialect.	Framework analysis	Moderate
Jagoe *et al*., 2007 [40]	India, Western Maharashtra	Rural	Bhagyalaxmi and Laxmi stoves	ARTI’s program, with support from Shell Foundation (since 2003)	FGDs at baseline and follow-up	FGDs with women users/non users.	May 2005–May 2006; language not specified	Framework analysis	Moderate
Pandey 1989 [38]	Central Nepal, Dhading District	Rural	Bikase stoves	Nepal Community Forestry Development Project (since 1984)	SSIs and PO	SSIs with 25 women (10 users; 15 non-users) from Brahmin and Cheetri castes	April–September 1987; Interviews in Nepali	Not stated; descriptive narrative	Moderate
Person *et al*., 2012 [34]	Western Kenya	Rural	Upesi Jiko charcoal stoves	Safe water and AIDS program, (SWAP) (since 2008)	SSIs	SSIs with 40 women (30 purchasers, 10 stove promoters)	July 2008–March 2009; interviews in Dholou	Modified grounded theory approach	Strong
Sesan (2012) [35]	Kenya, West Kochieng	Rural/ Urban **	Mainly upesi Jiko charcoal stoves with/without eaves space	USEPA project (2009–2010)	SSIs and PO	No. = 24 (15 women users and 9 elite interviews)	November–December 2009; language not reported	Not stated; descriptive narrative	Moderate
Simon (2007) [32]	India, Western Maharashtra	Rural	Bhagylaxmi, Laxmi cement stoves and other models	ARTI’s CBFCDprogram, with support from Shell Foundation (since 2003)	SSIs, KIIs and PO	No. = 55 (40 women users, 13 male + 2 female stove builders), KIIs (4 NGO employees and 7 field officers)	September–December 2005; interviews in English or Marathi	Not stated; descriptive narrative	Strong
Sovacool and Drupady (2011) [31]	Bangladesh	Rural/ Urban	Clay stoves	Intervention promoted by the company Grameen Shakti (since 2006)	Case study with use of SSIs	No. = 48 (among users, rural community leaders, manufacturers and 19 institutions)	June 2009–October 2010; Interviews in Bengali with local variation and dialects	Narrative analysis	Moderate
Troncoso *et al*., (2007) [37]	Mexico, Michoacán State	Rural	Patsari stoves	Regional program implemented by GIRA (2003–2006)	SSIs and PO	No. = 67 women (52 users, 15 non-users)	One year after stove installation; language not reported	Not stated; descriptive narrative and tables	Moderate
Troncoso *et al*., (2011) [33]	Mexico, Michoacán State	Rural	Patsari stoves	Regional program implemented by GIRA (2003–2006)	KIIs	No. = 24 (several categories of stakeholders including stove builders and project technicians)	At the end of program implementation; language not reported	Not stated; descriptive narrative	Moderate
Velasco (2008) [41]	Mexico, Michoacán State	Rural	Patsari stoves	Regional program implemented by GIRA (2003–2006)	Case study base on SSIs and PO	No. = 24 women users of one or multiple cooking technologies	6 weeks in 2008; language not reported	Not stated; descriptive narrative	Moderate

FGDs: Focus Group Discussions; KIIs: Key Informant Interviews; PO: Participant observation; SSIs: Semi-Structured Interviews; *: Only qualitative elements of mixed-methods studies are reported in this table; **: Study conducted in a setting defined as “peri-urban”; **^#^**: Quality appraisal adapted by Harden *et al*., 2009 and based on selected criteria; *Strong*: all or the majority criteria fulfilled; *Moderate*: most criteria fulfilled; *Weak*: few criteria fulfilled.

#### 3.3.3. Geography and Climate

Setting is particularly important in relation to fuelwood access and climate conditions (e.g., rainy and cold settings). IS that also meet heating needs can be popular in cold climates [30,41].

“*The (IS) stays warm for a long time. Some people, when they come and visit us they say, oh your home is so warm. […] The (traditional) stove cannot keep warm for a long time*” [30].

Where households reported walking long distances for fuel, and where the climate is problematic, there was an incentive to adopt an IS which requires less fuel [36,37,43]. Conversely, where biomass was freely available close to home, there was less inclination to adopt [35,37]. As Sesan notes:

“*Where people can still gather biomass freely in substantial quantities, the incentive to save on fuel cost is much less and (households) would not prioritise an improved stove.”* [35].

### 3.4. Domain 3: Knowledge and Perceptions

#### 3.4.1. Smoke, Health and Safety

Most women appreciated short-term health benefits following IS introduction including a reduction in burns [32,37,39,40], respiratory symptoms and other conditions such as headaches, runny nose, sore throat and sore eyes [31,34,36,37,39,40,43].

“*Now my arms don’t get burned. When I would put the wood in the stove it would burn my arm. Sometimes it would burn my forearm and upper arm.”* [39].

“*There was irritation in the throat, burning in the throat, that has become less. We are saved from this trouble by this stove*.” [36].

One woman also reported needing to seek less healthcare as a result of less smoke, and highlighted significant savings:

“*It is expensive when I go the doctor for sore throat reasons due to the smoke. The other day I paid 1300 pesos ($130 USD) including the ride to Zamora (city located close by) to visit the doctor*.” [41].

However, in the Mongolian study, users continued to have health concerns about the use of poor-quality coal but were less clear about the negative longer-term health effects associated with traditional practices.

“*It’s difficult to say that this (new) stove has a positive health effect. […] I don’t know exactly how it affects our health*.” [30].

An additional finding from two Indian studies was that women missed the effects of the smoke as a way of insect control [32,43].

Safety issues in relation to wood collection were reported as another important issue, with reduced fuel requirements putting women less at risk:

“*Sometimes due to the fear of the rangers we have to walk fast and we start breathing hard and the stomach starts aching [...]. I get hurt by the axe, snake bites, neck pains, thorns prick us, arms and legs pain and rangers harass us. Sometimes when we are caught, they take away our axe and then ask for money”* [43].

#### 3.4.2. Cleanliness

Following IS introduction, a cleaner kitchen was reported by users, [30,37,39] making the home environment more appealing [34]. This was an important incentive.

“*It produces less smoke and soot. The old one produced a lot of soot. Now it’s not like that—it’s so good.”* [30].

#### 3.4.3. Family Life and Social Aspects

Many women associated the IS with higher social status [34,39], Furthermore, the IS was reported to enable families to gather in the kitchen and socialise [39,43].

“*Now we can eat (our meals) in the kitchen easily because the smoke does not irritate us as it goes out through chimney*” [43]

The experience of neighbours and relatives was also reported to impact on the decision to adopt a stove in positive [34,43] or negative ways [38].

“*We all used the new stove but one person stopped using it […]. Now he is using his new stove because we encouraged him to use the new stove and tell him its qualities so he is using it now regularly*” [43].

#### 3.4.4. Tradition and Culture

It is important that the IS allows women to continue their traditional cooking practices. Sometimes problems emerged with cooking for large gatherings [34,37,38,39] such as burners being too small for large pots [38,39] and difficulties with lifting heavy pots onto the raised cooking surface [39]. Similarly, not feeling the need to change cooking habits, [31,33,36,37] not being able to cook traditional foods [38,40], and not being familiar with the IS [31] were barriers to adoption. As reported by an implementing NGO:

“*People have been cooking with traditional stoves for thousands of years, and those stoves need big pieces of firewood, many do not think a smaller, more efficient stove with smaller pieces of wood can actually cook the same*” [31].

### 3.5. Domain 4: Financial, Tax and Subsidy Aspects

#### 3.5.1. Stove Costs

Stove cost emerged as an important determinant of adoption even though cost varied greatly between studies. Stoves could be disseminated for free [33,37,38] subsidised [36,40,43] or purchased at full price [34,35]. Some users did not consider the IS a worthwhile investment when mud or clay stoves could be produced free of charge [32,34,36], especially given competing household priorities [34,35]. One woman suggested that with training, women would be able to produce the stoves themselves at no cost:

“S*how me how to make it once and I will make it for free. Why does someone need to make it for me?*” [32].

Some families however, could afford an IS and clearly recognised its benefits:

“*We purchased it for 300 rupees [...]. It is not expensive because this stove has many benefits and its smoke goes out directly*” [43].

A Mexican programme, where IS were provided free, offered contrasting insights: some programme participants said they were less likely to adopt a free IS than one they paid for, as they valued the purchased IS much more [33].

#### 3.5.2. Payment Modalities

Households typically have low incomes and limited access to credit, and more pressing needs to satisfy, in particular ensuring sufficient quantities of food [34,35]. In many households, the decision to adopt requires planning and negotiations about how to allocate scarce resources.

“*So many people are longing to have the cookstove but the price is so expensive. People cannot afford it now with the drought. The hunger has hit us hard*” [34].

Some market-based projects in India used payment in instalments and village-level subsidies [32,40,43]. While these were generally well-received and facilitated adoption, some users suggested that they would prefer to use savings and purchase the stove with a single payment [40]. In Kenya, community lending schemes were also found to favour adoption [34].

### 3.6. Domain 5: Market Development

#### 3.6.1. Demand Creation

Householders are often unaware of the detrimental health and environmental effects of smoke from traditional cooking practices, suggesting a need for awareness raising about health and broader benefits including fuel and time savings [30]. Mechanisms used to promote awareness include stove demonstrations at home and in the market [32,34], use of community meetings or door-to-door promotion by health promoters [34,35].

“*Many people come to my home to see the upesi jiko and because of the good things they see about it, this makes them like it […]. Observing it is what makes people like it and want to buy it*.” (Health promoter) [34].

Some projects provide incentives for adoption. In Maharashtra state (India), for example, the government offered awards, prompting members of the community to exert pressure on each other.

“*The neighbours don’t want us to use this [traditional] stove. Not because of the smoke. They say it prevents us from winning a “Clean Village” award from the State Government*” [32].

#### 3.6.2. Business and Sales Approach

For stove builders, there is potential for business growth but this is accompanied by uncertainty about demand [32]. In an Indian study, stove builders reported anxiety related to enterprise sustainability, inability to plan for large-scale production and balancing high-quality craftsmanship with administrative and sales tasks [32].

“*It takes 5–6 days to make one batch of chulah with the mixing, casting, drying and settling. It’s not easy to find customers and properly build chulah*” [32].

In another study in Bangladesh, staff retention and job satisfaction were also reported as an issue:

“*People don’t think that becoming an IS technician is a dignified job, with no transferable skills, and many men are reluctant to embrace an organization associated with women’s empowerment*” [31].

In an Indian study, female participation in self-help groups was encouraged in brokering agreements between village members, local government and the NGO, to purchase improved chulhas with loans or instalments [32]. This approach was found to be useful in increasing uptake.

“*We had to propose a loan plan that was best for everyone. Then we brought a representative from ARTI to help us to show the improved stove to villagers and members of the panchayat (village council). Some were not easy to convince but after some time the villagers, panchayat and ARTI began to like the idea*” [32].

#### 3.6.3. Supply Chain

One advantage of building stoves locally is that there is no reliance on a complex supply chain [31], and it also provides opportunities for local income generation. Another reported advantage is that villagers trust the stoves more. Indeed, one study reported that villagers sometimes mistrust stoves that are not built locally [32]. Another barrier to dissemination among smaller communities is the use of expensive materials or needing to buy materials to produce small numbers:

“*I buy materials only for 10 to 15 chulah (stoves), which increases the price for material. The price of chulah also increases*” [32].

### 3.7. Domain 6: Regulation, Legislation and Standards

No factors reported for this domain.

### 3.8. Domain 7: Programmatic and Policy Mechanisms

#### 3.8.1. Institutional Arrangements and Community Involvement

For some projects, administrative arrangements and donor requirements [33,35] may work against market sustainability. In particular, short term projects may not focus on sustainable use of improved technologies [35] and bureaucracy may make it difficult for the community to continue stove production [33].

“*I would have liked to continue building the IS because it’s a good business but the NGO people offered to me the mould once I had built 50 stoves. Because I had only built 42, I didn’t have the mould anymore*” (Stove builder) [33].

A good understanding of local views prior to developing a marketing strategy was stressed [33]. Here, the donor required precise numbers of stoves to be fitted, and as a result, the project workers felt they were persuading rather than informing the community:

“*It was not a question of considering the opinions of the users, but to convince them. As I was asked to bring a number of clients, I had to think what to tell them, in order to convince them*” [33].

This lack of proper partnership between project donors, project workers and stove users emerged as a barrier to creating sustainable change.

#### 3.8.2. User Support and Post-Acquisition Services

User training, especially during the first two weeks after installation, could be an important factor for regular use [37], since it allows recognition and resolution of early problems with the new technology.

“*We had a problem of its fitting, and we didn’t know how to clean its chimney - when men came to install it they told us everything and our problems have been solved*” [43].

One means of ensuring this is to provide women themselves with the skills they need. In a Mexican study, many women stated that they would be happy to reduce their reliance on outside technical support.

“*I would like to know on my own how to fix a comal—if it broke or has some other problem, and how to repair my stove so that I am not left without a stove*” [39].

## 4. Discussion

### 4.1. Factors Influencing Uptake

This is the first qualitative systematic review to investigate factors influencing uptake of IS in low- and middle-income countries. Overall, the review identified 18 factors from qualitative studies, across six domains (D1—four factors, D2—three factors, D3—four factors, Domain 4—two factors, Domain 5—three factors, Domain 7—two factors), while the domain on “Regulation, legislation and standards” remained unpopulated. This is partly a reflection of the available qualitative evidence, which tends to focus more on the user than on other stakeholders involved in IS production and dissemination. Each factor, when present, may serve as an enabler or, when absent, may serve as a barrier to uptake. Some factors primarily act at household or community level (e.g., Household and settings; Knowledge and perceptions) whereas other factors primarily act at regional, national and even international level (e.g., Financial, tax and subsidy aspects; Regulation, legislation and standards). Also, it seems likely that factors within and across domains and levels are interrelated and jointly influence uptake in additive, or synergistic ways.

This review highlights the importance of user perspectives and other key actors in the design and marketing of IS programmes; if users do not value the stoves and/or the stoves do not meet their needs, a programme is unlikely to be successful. More specifically, a stove design that allows users to continue cooking traditional dishes using traditional pots and cooking utensils is important for adoption and sustained use. Stoves that result in fuel and time savings are also valued, but the need to prepare and chop wood more carefully and the inability to burn other forms of biomass can be a disadvantage, especially for poorer households. The cost of stoves is also an important factor, but ability to pay for the stove does not alone guarantee use over time. Extra cleaning and maintenance and use of poor-quality materials also impacts on sustained use, although women appreciate the health benefits of having less smoke and a cleaner home environment.

Thus, incorporating users’ views is an important part of the success of any programme. Stove demonstrations in the home or the community, the use of social networks, and community incentives show some success in increasing demand. User training and technical support are also needed post acquisition to support change and avoid initial frustration with the new technology. In addition, meaningful involvement of the community in the setting up and running of any project is important for short-term success as well as longer-term sustainability.

However, the focus of qualitative findings on user perspectives is not necessarily a reflection of the potential utility of qualitative methods in exploring factors operating at a broader scale, but probably a reflection of the tendency for qualitative methods to be used within smaller-scale projects. Using qualitative methods to explore factors impacting at regional, national or even international level including market development and policy mechanisms would be useful in terms of understanding the impact and interplay of different factors across all domains and levels. Thus qualitative methods could be used to explore and understand the perspectives of other relevant stakeholders, including regional and national policy makers, project implementers, NGOs and stove makers for example.

### 4.2. Equity Considerations

Reliance on traditional household energy practices is highly unequally distributed, with rural and socio-economically deprived groups being most dependent on traditional practices and with women and children suffering disproportionately greater health risks relative to men [44]. No projects were found that specifically considered equity. Nevertheless, three equity dimensions could be identified across the studies: gender, socio-economic status and geographical location (rural *vs.* urban).

There is evidence that women and children stand to gain most if families can be encouraged to acquire an IS. The possibility of scaling up programmes that harness the role of women is a promising development. New cooking technologies have the potential to advance women’s prospects and involve them actively in the stove market in a host of different ways through involving them in promotion and marketing. This could benefit them economically and support financial sustainability [45]. However, household decision-making practices sometimes mean women have little say in the decision to purchase. Thus, it is particularly important to target both men and women in the community when creating market demand.

More work is required to examine how IS programmes can be designed to address the needs of socio-economically disadvantaged groups. This relates to ways of reaching poor households and communities with information and marketing strategies, and also to the feasibility of subsidies, micro-credit schemes and payment by instalments. The argument, based on Roger’s Diffusion of Innovation Theory [42], that uptake is more likely to succeed among better-off communities and subsequently facilitate change in poorer communities, also merits scrutiny. This issue is linked to market considerations since, if projects are to be sustainable, they need a successful market and therefore, sellers tend to target better-off segments of society. However, as stoves become more advanced, this may put them further out of reach of low-income households. In theory, large-scale production should reduce price (and improve quality), while innovations in financing for both suppliers and potential consumers could be effective in extending access.

Rural and urban communities may also require different approaches, since findings are consistent with there being less incentive in rural areas to save fuel through purchasing a new stove. This is because the opportunity cost of fuel collection and the value attached to fuel savings can vary substantially in urban and rural settings, and depending on fuel availability.

As stated by the Commission on Social Determinants of Health [46] “Competent, regular health equity impact assessment of all policy-making and market regulation should be institutionalized nationally and internationally.” IS programmes are no exception. Equity is critical in relation to the scaling up of interventions at global (*i.e*., making sure that the most affected countries are reached) and national level (*i.e*., making sure that the most disadvantaged households are reached), and therefore equity with respect to gender, socio-economic status and urban-rural location should receive special consideration during policy and programme development.

### 4.3. Strengths and Limitations

The review used a comprehensive search strategy to identify all available qualitative evidence meeting the inclusion criteria. In particular, grey literature searches uncovered evidence from reports and dissertations not accessible through the peer-reviewed literature. However, whilst efforts were made to include foreign language evidence (studies published in six languages were considered) the Chinese databases were not screened and could represent a potentially rich source of additional information.

The review followed strict standards for screening, data extraction and quality appraisal of studies. For the latter, we adopted established methods to examine quality appraisal [26]. In practice, it was sometimes difficult to apply the criteria due to lack of information that would allow a reliable distinction to be made between poor quality data collection and analysis *versus* inadequate reporting of methods and findings. This problem has been described previously in relation to appraisal of qualitative evidence in systematic reviews [47]. With all this in mind we erred on the side of caution, and where information was not available, we did not ascribe a score to the item. Thus our appraisal scoring can be considered to be conservative; nevertheless, all but one study received a moderate or strong quality score. For future studies however, it will be important to address both quality in relation to data collection, and in reporting. The broader review reported elsewhere [48] identifies many of the factors identified here based on quantitative and case study findings, thereby strengthening the overall validity of these findings. In addition, it provides evidence in relation to the less populated domains of our framework.

Finally, thematic synthesis was employed within the analysis, and factors identified were compiled based on a comprehensive a-priori framework. Themes were identified without reference to the framework to allow themes to be generated from the data without restricting the researcher to pre-specified categories. Subsequently, when the identified themes were applied to the framework, this proved to be a useful conceptual tool within which to organise the findings.

In summary, this review has provided in-depth insights of the perceptions of actual and potential users of improved stoves and other stakeholders involved in building, marketing or selling improved stoves. It has also reported detailed findings in relation to equity. It is important to remember that some of the findings are context-dependent (e.g., culture, setting, programmatic approach) and may not be easily transferable to other contexts. At the same time, many findings are common in quite different contexts and settings, suggesting that they may act more or less universally as barriers and enablers. This ability to explore factors in context, in many ways, can be viewed as a strength of a qualitative study design, but we would also advocate the need for further studies with different types of IS, and in different contexts and settings, including urban settings, in order to strengthen the current evidence base.

Adoption and sustained use of improved stoves to improve long-term health is clearly dependent on stove effectiveness, an issue not explicitly considered in this review, primarily because the included studies do not report effectiveness. Where IS are not effective in delivering a reduction in HAP that is apparent to users, this may have a negative effect on uptake and could adversely impact on a decision to invest in the future or on other community members adopting the technology.

## 5. Conclusions and Policy Implications

Achieving adoption and sustaining use of improved solid fuel stoves is a complex and challenging issue. Since factors within and across domains and at different levels interact, this suggests that the connection between household, community, programme and societal levels is important, if programmes are to be successful at scale and over extended periods of time. This review demonstrates the relevance and potential of qualitative approaches in informing programmatic and policy decisions. In addition, based on a range of study designs, the broader systematic review [48,49] provides further detailed evidence across all domains, and should be reviewed carefully by all relevant organisations working in the field of IS scale up.

The lack of funding available for implementation research, and the division between those who implement interventions (*i.e*., governmental or non-governmental organisations in developing and middle-income countries who may lack the capacity to conduct in-depth evaluation) and those who conduct research (*i.e*., researchers who are more likely to be interested in and receive funding for rigorous research designs focused on health issues that may not inform better understanding of the success or failure of implementation) remains an issue. Implementation projects that integrate high-quality interdisciplinary research and evaluation incorporating qualitative and quantitative methods can provide more valid and reliable evidence on which to base future scale up efforts.

In conclusion, there is a need for an upfront, comprehensive research agenda to accompany large-scale initiatives promoting IS dissemination. This should increase the range of perspectives, involving all major stakeholders including beneficiaries, civil society, government and the private sector. Within this research agenda, there is a clear need for quantitative assessments of effectiveness to be complemented by carefully devised and conducted qualitative studies. These should focus on assessing factors that determine adoption and sustained use, as well as where appropriate, exclusive use including various dimensions of equity such as gender, socio-economic status and urban-rural setting. Qualitative research is particularly valuable in exploring stakeholder perspectives and the importance of a wide range of stakeholder views should not be overlooked by those responsible for developing and implementing IS programmes.
